# Electroacupuncture-like stimulation at Baihui and Dazhui acupoints exerts neuroprotective effects through activation of the brain-derived neurotrophic factor-mediated MEK1/2/ERK1/2/p90RSK/bad signaling pathway in mild transient focal cerebral ischemia in rats

**DOI:** 10.1186/1472-6882-14-92

**Published:** 2014-03-07

**Authors:** Chin Yi Cheng, Jaung Geng Lin, Shan Yu Su, Nou Ying Tang, Shung Te Kao, Ching Liang Hsieh

**Affiliations:** 1 School of Chinese Medicine, College of Chinese Medicine, China Medical University, Taichung 40402, Taiwan; 2 Department of Chinese Medicine, Hui-Sheng Hospital, Taichung 42056, Taiwan; 3 Acupuncture Research Center, China Medical University, Taichung 40402, Taiwan; 4 Department of Chinese Medicine, China Medical University Hospital, Taichung 40447, Taiwan; 5 School of Post-baccalaureate Chinese Medicine, College of Chinese Medicine, China Medical University, Taichung 40402, Taiwan; 6 Graduate Institute of Integrated Medicine, College of Chinese Medicine, China Medical University, Taichung 40402, Taiwan

**Keywords:** Electroacupuncture, Brain-derived neurotrophic factor, Phospho-ERK1/2, Phospho-p90RSK, Phospho-Bad, Apoptosis

## Abstract

**Background:**

This study was designed to evaluate the effects of electroacupuncture-like stimulation at Baihui (GV20) and Dazhui (GV14) acupoints (EA at acupoints) following mild cerebral ischemia-reperfusion (I/R) injury. Furthermore, we investigated whether brain-derived neurotrophic factor (BDNF)-mediated activation of extracellular signal-regulated kinase (ERK)1/2 signaling pathway is involved in the neuroprotection induced by EA at acupoints.

**Methods:**

Rats were subjected to middle cerebral artery occlusion (MCAo) for 15 min followed by reperfusion for 3 d. EA at acupoints was applied 1 d postreperfusion then once daily for 2 consecutive days.

**Results:**

Following the application of EA at acupoints, initiated 1 d postreperfusion, we observed significant reductions in the cerebral infarct area, neurological deficit scores, active caspase-3 protein expression, and apoptosis in the ischemic cortex after 3 d of reperfusion. We also observed markedly upregulated BDNF, phospho-Raf-1 (pRaf-1), phospho-MEK1/2 (pMEK1/2), phospho-ERK1/2 (pERK1/2), phospho-90 kDa ribosomal S6 kinase (pp90RSK), and phospho-Bad (pBad) expression, and restored neuronal nuclear antigen (NeuN) expression. Pretreatment with the MEK1/2 inhibitor U0126 abrogated the effects of EA at acupoints on cerebral infarct size, neurological deficits, active caspase-3 protein, and apoptosis in the ischemic cortex after 3 d of reperfusion. Pretreatment with U0126 also abrogated the effects of EA at acupoints on pMEK1/2, pERK1/2, pp90RSK, pBad, and NeuN expression, but did not influence BDNF and pRaf-1 expression.

**Conclusion:**

Overall, our study results indicated that EA at acupoints, initiated 1 d postreperfusion, upregulates BDNF expression to provide BDNF-mediated neuroprotection against caspase-3-dependent neuronal apoptosis through activation of the Raf-1/MEK1/2/ERK1/2/p90RSK/Bad signaling cascade after 3 d of reperfusion in mild MCAo.

## Background

Cerebral ischemia-reperfusion (I/R) injury produces large amounts of reactive oxygen species, which initiate a series of cellular events and that lead to necrosis and apoptosis [1]. In mild transient focal cerebral ischemia, brain infarction can develop and progress in a delayed manner, and become grossly visible after 3 d of reperfusion [2,3]. Apoptosis, which is dependent on caspase-3 activation, plays a significant role in the pathology of the delayed infarction and predominates in ischemic neurons during mild focal cerebral ischemia [2-4]. Neurotrophic factors provide neuroprotection against caspase-3-dependent apoptosis by activating various signal transduction pathways following cerebral I/R injury [5,6].

Brain-derived neurotrophic factor (BDNF) is a member of the neurotrophin family that plays an important role in neuroplasticity, neuron development, differentiation, and neuronal survival [7-9]. It binds to the specific tyrosine kinase B (TrkB) receptor on neurons to activate two major intracellular signal transduction pathways: the phosphatidylinositol 3-kinase (PI3K) and the mitogen-activated protein kinase (MAPK) pathways [10]. Koh identified that the MAPK/extracellular signal-regulated kinase (ERK)1/2 signaling pathway is a critical mediator of neuronal cell survival against apoptosis in a focal cerebral ischemia model in rats [11]. A number of studies have shown that BDNF provides neuroprotective effects against apoptotic cell death through the stimulation of a protein kinase cascade that includes the sequential activation of Raf-1, MAPK/ERK kinase1/2 (MEK1/2), and ERK1/2 [12,13]. Extracellular signal-regulated kinase1/2 then phosphorylates the 90 kDa ribosomal S6 kinase (p90RSK), leading to the phosphorylation of Bad and the attenuation of caspase-3-dependent apoptosis [14,15]. In previous studies, BDNF agonists improved neurological function and reduced infarct size in a transient focal cerebral ischemia model in rats [16,17]. However, MEK/ERK inhibitors abrogated BDNF-induced neuroprotection in hippocampal neurons in vitro [6] and in neonatal hypoxic-ischemic brain injury in vivo [18].

Chinese physicians have used acupuncture to treat various disorders for several centuries [19]. According to traditional Chinese medicine, Baihui (GV20) and Dazhui (GV14) are both acupoints on the “Du meridian”, which travels into the brain, and are commonly used to treat stroke. Experimental studies in rats have shown that EA stimulation at acupoints (such as Baihui and Shuigou acupoints) can attenuate cerebral infarction and improve neurological outcome after transient middle cerebral artery occlusion (MCAo) [20,21]. Kim et al. have reported that pretreatment with EA at Baihui and Dazhui acupoints elicit neuroprotection through increased BDNF and stromal cell derived factor-1α (SDF-1α) expression 1 d after MCAo [22]. Other studies have also shown that EA can potentially provide neuroprotection against cerebral ischemic insults through activation of various survival signaling pathways [20,23-25]. However, the detailed mechanisms underlying BDNF-induced neuroprotection resulting from EA stimulation at Baihui and Dazhui acupoints following mild cerebral I/R injury remain unclear. The aim of this study was, therefore, to evaluate the effects of EA-like stimulation at Baihui and Dazhui acupoints (EA at acupoints) after 15 min of ischemia followed by 3 d of reperfusion, and to elucidate the mechanisms involved in the BDNF-mediated signaling transduction pathway.

## Methods

### Experimental animals

Male Sprague Dawley (SD) rats weighing 300 g to 350 g were used. This study was reviewed and approved by China Medical University Institutional Animal Care and Use Committee (Permit Number: 100-215-c), and the committee recognized that the proposed experimental procedures compiled with the Animal Protection Law by the Council of Agriculture, Executive Yuan, Taiwan. All the procedures with animals avoided or minimized discomfort, distress, and pain to the animals.

### The MCAo model

The MCAo model was established in the SD rats using an intraluminal suture method as described previously [26]. Briefly, the rats were anesthetized with chloral hydrate (400 mg/kg, intraperitoneally), and the right common carotid artery (CCA) and internal carotid artery (ICA) were exposed by way of an incision in the midline neck prior to ligation of the pterygopalatine artery close to its branch. A 3–0 nylon filament suture, blunted at the tip by a flame and coated with poly-L-lysine (Sigma, USA), was inserted into the right external carotid artery (ECA) through the CCA into the ICA for a distance of 20 mm to 25 mm to block the origin of the middle cerebral artery (MCA). The suture was removed slowly to reestablish the blood flow after 15 min of MCAo. The rectal temperature of the rats was maintained at 37 ± 0.5°C throughout the experimental procedure using an electrical heating pad.

### Electrode implantation

Following the completion of the MCAo operation, the rat’s head was fixed to the stereotactic frame and its scalp or costal skin was incised. The electrode consisted of 0.5-mm stainless steel wires used for acupoint (or nonacupoint) stimulation. It was implanted in Baihui (midpoint of the parietal bone, 4-mm depth of insertion forward) and Dazhui (below the spinous process of the seventh cervical vertebra, 5-mm depth of insertion vertically) acupoints, or in bilateral costal regions (nonacupoints). The rat was then returned to the cage.

### Assessment of neurological status

The neurological status of each rat was assessed after 1 d and 3 d of reperfusion. Motor, sensory, balance, and reflex functions were determined using the modified neurological severity score as described previously [27]. The neurological function of each rat was graded using a numeric scale from 0 to 18. (normal score, 0; maximal deficit score, 18). Excepting the sham-operation group, rats with neurological deficit scores equal to or greater than 7 after 1 d of reperfusion were included in further analyses, whereas rats with neurological deficit scores less than 7 were excluded from subsequent analyses.

### Experiment A

#### *Grouping*

Rats were randomly divided into 6 groups (n = 5 or 6): the EA-like stimulation at acupoints (EA group), EA-like stimulation at nonacupoints (non-acup), model, sham-operation (sham), treatment with U0126 in the EA (U0126 + EA) and treatment with vehicle in the EA (vehicle + EA) groups. Rats in the EA group were subjected to 15 min of MCAo. After 1 d of reperfusion, rats received EA at acupoints once daily for 2 consecutive days. Rats were then sacrificed after 3 d of reperfusion. Rats in the non-acup group were subjected to the same procedure as rats in the EA group but received EA at nonacupoints. Rats in the model group were subjected to the same procedure as rats in the EA group but did not receive EA. Rats in the sham group were subjected to the same procedure as rats in the model group but the MCA origin was not occluded. Rats in the U0126 + EA group were subjected to the same procedure as rats in the EA group but also received an intracerebroventricular (ICV) injection of the MEK1/2 inhibitor U0126 30 min prior to the onset of EA at acupoints. Rats in the vehicle + EA group were subjected to the same procedure as rats in the EA group but also received an ICV injection of the vehicle 30 min prior to the onset of EA at acupoints.

#### *Intracerebroventricular injection of U0126 or vehicle*

Rats were anesthetized with a 2% isoflurane/oxygen mixture and an ICV injection of a 4 μl solution containing U0126 (4 μg in vehicle, #662005 Calbiochem) or vehicle (DMSO diluted in saline) was administered to the right hemisphere. Injections were performed using a Hamilton syringe with a 26 gauge needle (Hamilton Company, Nevada, USA). The location of each injection was 0.8 mm posterior to the bregma, 1.5 mm lateral to the midline, and 3.5 mm deep into the skull surface.

#### *Electroacupuncture-like stimulation at Baihui and Dazhui acupoints or nonacupoints*

An EA apparatus (Trio 300, ITO Co., Germany) was used to generate EA at acupoints or nonacupoints for 25 min once daily for 2 consecutive days. The stimulation parameters were 5 Hz amplitude-modulated wave, 2.7 mA to 3.0 mA intensity, and 150 μs pulse width. The rats were awake and moving freely in the cage during EA at acupoints or nonacupoints.

#### *Measurement of cerebral infarct area*

Following their neurological status evaluations after 3 d of reperfusion, the rats were sacrificed under deep anesthesia. The brains were removed immediately and cut into 2-mm sections using a brain matrix. The sections were then stained with 2% 2,3,5-triphenyltetrazolium chloride (TTC; Merck, Germany) for 15 min at 37°C. Brain tissue was differentiated according to staining: white for infarct area and red for noninfarct area. The cerebral infarct areas of the first 6 sections from the frontal lobe were measured using image analysis software (ImageJ, Java). The ratio of infarct area to total brain area was also calculated.

### Experiment B

Rats were randomly divided into 5 groups: EA, non-acup, model, sham and U0126 + EA groups. They were then subjected to the experimental procedure described in Experiment A.

#### *Immunohistochemical (IHC) analysis*

After 3 d of reperfusion and 15 min of cerebral ischemia, rats were sacrificed under deep anesthesia (n = 5 or 6). Rats were transcardially perfused with 200 ml 0.9% saline and 200 ml 4% paraformalaldehyde (PFA; pH 7.4). Rat brains were removed quickly and postfixed in 4% PFA followed by 30% sucrose (weight/volume) for 3 d, after which they were cut into 15-μm sections using a cryostat. Brain sections were rinsed with Dulbecco’s phosphate buffered saline (DPBS; Sigma-Aldrich) containing 0.01% Tween-20 and immersed in 3% hydrogen peroxide (H_2_O_2_)/methanol for 15 min to inhibit endogenous peroxidase activity. They were then incubated with a 10% normal animal serum (ScyTek, Logan, Utah, USA) for 20 min at room temperature (RT) before incubation in moist chambers with a rabbit anti-BDNF (1:500 dilution, AB1779 Millipore), rabbit anti-phospho-Raf-1 (pRaf-1) (1:100 dilution, sc-28005-R Santa Cruz), rabbit anti-phospho-MEK1/2 (pMEK1/2) (1:200 dilution, #2338 Cell Signaling Technology), rabbit anti-phospho-ERK1/2 (pERK1/2) (1:200 dilution, #4376 Cell Signaling Technology), or rabbit anti-phospho-p90RSK (pp90RSK) (90 kD, 1:250 dilution, #9344 Cell Signaling Technology) antibody overnight at 4°C. Following incubation with the appropriate secondary antibody and avidin-biotin peroxidase complexes (ABC kit, ScyTek, Logan, Utah, USA), sections were colored using a 3,3′-diaminobenzidine (DAB) kit (ScyTek, Logan, Utah, USA), and counterstained with hematoxylin. The stained sections were mounted in mounting media (Assistant-Histokitt, Germany) and immunopositive cells were detected using microscopic analysis (Axioskop 40, Zeiss). Negative controls for BDNF, pRaf-1, pMEK1/2, pERK1/2, and pp90RSK staining were prepared using adjacent serial sections from the EA group incubated without primary antibodies.

#### *Immunohistochemical costaining*

Brain sections were immersed in 3% H_2_O_2_/methanol for 15 min and then incubated with a diluted normal blocking serum (Vector Laboratories, CA, USA) at RT for 25 min. Sections were then incubated with a mouse antineuronal nuclei (NeuN) antibody (1:200 dilution, MAB 377 Chemicon) 1.5 h at 37°C and washed with DPBS. Following their incubation with the diluted biotinylated secondary antibody and an ABC-AP reagent (AK-5002, Vectastain), the sections were stained with an alkaline phosphatase substrate solution (SK-5300, Vector Blue). They were then incubated with a rabbit anti-active caspase-3 antibody (17 kD, 1:100 dilution, AB3623 Chemicon) for 1.5 h at 37°C and washed with DPBS. Following their incubation with the diluted biotinylated secondary antibody and an ABC-AP reagent (AK-5001, Vectastain), the sections were stained with an alkaline phosphatase substrate solution (SK-5100, Vector Red), dried, and mounted in mounting media (Assistant-Histokitt, Germany). Finally, the immunopositive cells were detected using microscopic analysis (Axioskop 40, Zeiss).

#### *Terminal deoxynucleotidyl transferase-mediated dUTP-biotin nick-end labeling (TUNEL) assay*

Terminal deoxynucleotidyl transferase-mediated dUTP-biotin nick-end labeling analysis was used to identify cells with nuclear DNA fragmentation in the ischemic cortex. Terminal deoxynucleotidyl transferase-mediated dUTP-biotin nick-end labeling staining was performed according to the manufacturer’s instructions (QIA33 Calbiochem, USA). Briefly, brain sections adjacent to those used in IHC analysis were incubated with 20 μg/ml proteinase K for 20 min at RT, rinsed with a Tris-buffered saline and incubated with a 1 × TdT equilibration buffer for 30 min at RT. They were then incubated with a TdT labeling reaction mixture for 1.5 h at 37°C. After addition of the stop solution and blocking buffer, sections were incubated with 1 × conjugate solution for 30 min at RT, and the TUNEL-positive cells were visualized using a DAB kit (Calbiochem). Finally, sections were counterstained with methyl green (Calbiochem).

#### *Western blot analysis*

Three days after reperfusion, the rats were anesthetized with choral hydrate (n = 4). The rat brains were then removed and sectioned coronally from -4.3 mm to +1.7 mm bregma. The brain was separated into the right cortex, right striatum, left cortex, and left striatum, and the right cortex was weighed and homogenized in an ice cold phosphate buffered saline (PBS) (0.5 ml). Lysates were centrifuged at 500 × g for 10 min at 4°C, and the supernatant was removed. After addition of 200 μl cytosol extraction buffer A (#K266-25 BioVision, USA) and 11 μl cytosol extraction buffer B (#K266-25 BioVision, USA), the suspension was centrifuged at 16000 × g for 30 min at 4°C. The supernatant was collected and saved as the cytosolic fraction. The protein concentration of the cytosolic fraction was determined using a Bio-Rad assay. The samples were boiled at 100°C in a sodium dodecyl sulfate (SDS) gel loading buffer for 10 min and loaded onto a 10% SDS polyacrylamide gel. After electrophoresis, the separated proteins were electrotransferred to a nitrocellulose membrane (Hybond-c Extra, Amersham Biosciences, UK) in transfer buffer. The membranes were incubated in 5% skim milk containing 0.1% Tween 20 for 60 min at RT to block nonspecific binding. They were then incubated with a rabbit anti-pMEK1/2 (1:1000 dilution, #2338 Cell Signaling Technology), rabbit anti-pERK1/2 (1:1000 dilution, #4376 Cell Signaling Technology), rabbit anti-pp90RSK (1:1000 dilution, #9344 Cell Signaling Technology), or rabbit anti-phospho-Bad (pBad) (1:1000 dilution, #9291 Cell Signaling Technology) antibody overnight at 4°C. The transferred membranes were also probed with a monoclonal antibody specific for actin (1:5000 dilution, MAB1501 Chemicon) as an internal control for the cytosolic fraction. After washing, membranes were incubated with an anti-rabbit horseradish peroxidase (HRP)-linked IgG (1:5000 dilution, Jackson ImmunoResearch), an anti-mouse HRP-linked IgG (1:5000 dilution, Santa Cruz Biotechnology), or a HRP-conjugated anti-biotin (1:5000 dilution, Cell Signaling Technology) antibody in a PBS for 1 h at RT. Proteins were detected using an enhanced chemiluminesence reagent kit (#34080 Thermo Scientific, USA) according to the manufacturer’s instructions. Densitometric analysis was performed using Alpha Innotech Analyzer software. The optical density was calculated and the levels of proteins were expressed as the densitometric ratio of proteins to actin.

#### *Statistical analysis*

Data are expressed as mean ± standard deviation (SD). All variables showed approximately normal distribution and parametric testing, such as analysis of variance (ANOVA), was appropriate. Data from all experimental groups were compared using one-way ANOVA followed by post-hoc analysis using the Scheffe’s test. A *P*-value *<* 0.05 was considered statistically significant.

## Results

### Effects of EA at acupoints on cerebral infarct area

**Figure 1:**
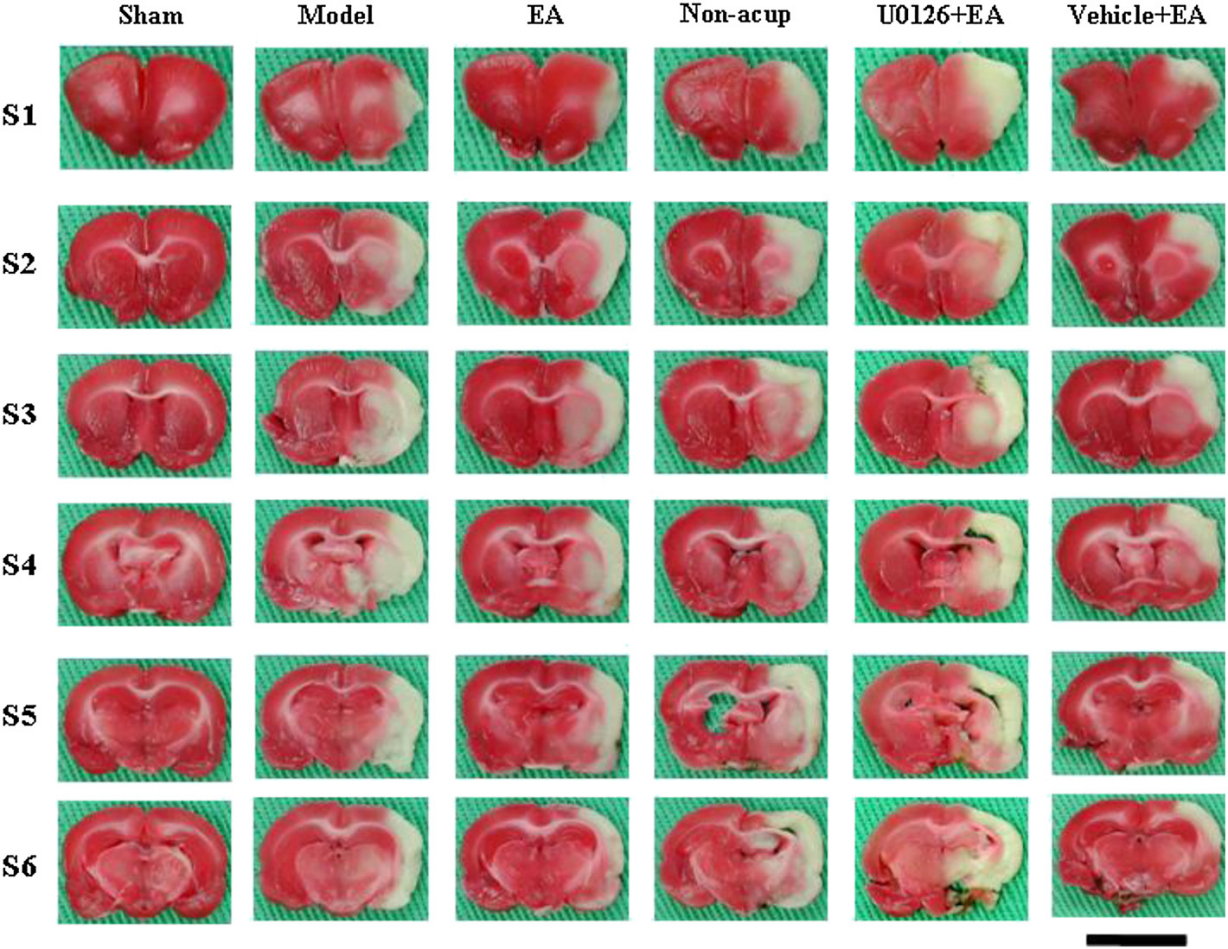
**Focal cerebral infarct areas (S1 to S6) among the experimental groups after 15 min of ischemia followed by 3 d of reperfusion (n = 4 to 6).** 2,3,5-Triphenyltetrazolium chloride staining showed the infarct area as white and the noninfarct area as red. Sham, sham group; Model, model group; EA, EA group; Non-acup, non-acup group; U0126 + EA, U0126 + EA group; Vehicle + EA, vehicle + EA group. Scale bar = 1 cm.

**Figure 2:**
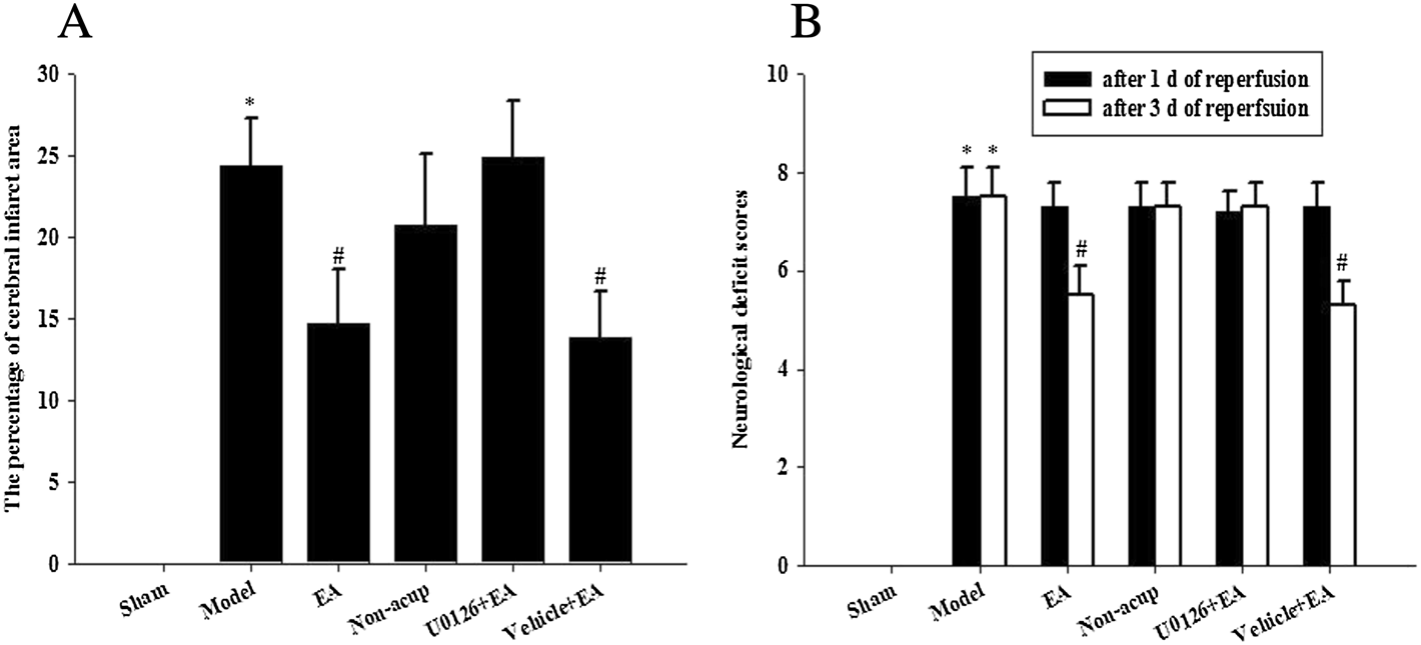
**Effects of EA at acupoints on cerebral infarct and neurological status. (A)** The percentage of cerebral infarct areas among the sham, model, EA, non-acup, U0126 + EA, and vehicle + EA groups were measured after 3 d of reperfusion. **(B)** The neurological deficit scores among the sham, model, EA, non-acup, U0126 + EA, and vehicle + EA groups were measured after 1 d and 3 d of reperfusion. Data are presented as mean ± SD. *P < 0.05 compared with the sham group; #*P* < 0.05 compared with the model group.

Rats developed cerebral infarct after 15 min of MCAo followed by 3 d of reperfusion (Figure 1). The percentage of cerebral infarct area was significantly higher in the model group than in the sham group (*P* < 0.05), and significantly lower in the EA and vehicle + EA groups than in the model group (both *P* < 0.05; Figures 1 and 2A). The percentage of cerebral infarct area among the model, non-acup, and U0126 + EA groups showed no significant difference (*P* > 0.05; Figures 1 and 2A), indicating that U0126 (MEK1/2 inhibitor), but not vehicle (solvent), pretreatment eradicated the effects that caused the significant differences in infarct area between the model and EA groups.

### Effects of EA at acupoints on neurological status

Rats developed neurological deficits after 15 min of MCAo followed by 1 d of reperfusion. Neurological deficit scores among the model, EA, non-acup, U0126 + EA, and vehicle + EA groups showed no significant difference (*P* > 0.05; Figure 2B). After 3 d of reperfusion, the neurological deficit scores were higher in the model group than in the sham group (*P* < 0.05). However, the neurological deficit scores were markedly lower in the EA and vehicle + EA groups than in the model group (both *P* < 0.05; Figure 2B). After 3 d of reperfusion, the neurological deficit scores in the model, non-acup, and U0126 + EA groups showed no significant difference (*P* > 0.05; Figure 2B), indicating that U0126 pretreatment eradicated the effects that caused the difference in the neurological deficit scores between the model and EA groups.

### Effects of EA at acupoints on BDNF, pRaf-1, pMEK1/2, pERK1/2, and pp90RSK expression

**Figure 3:**
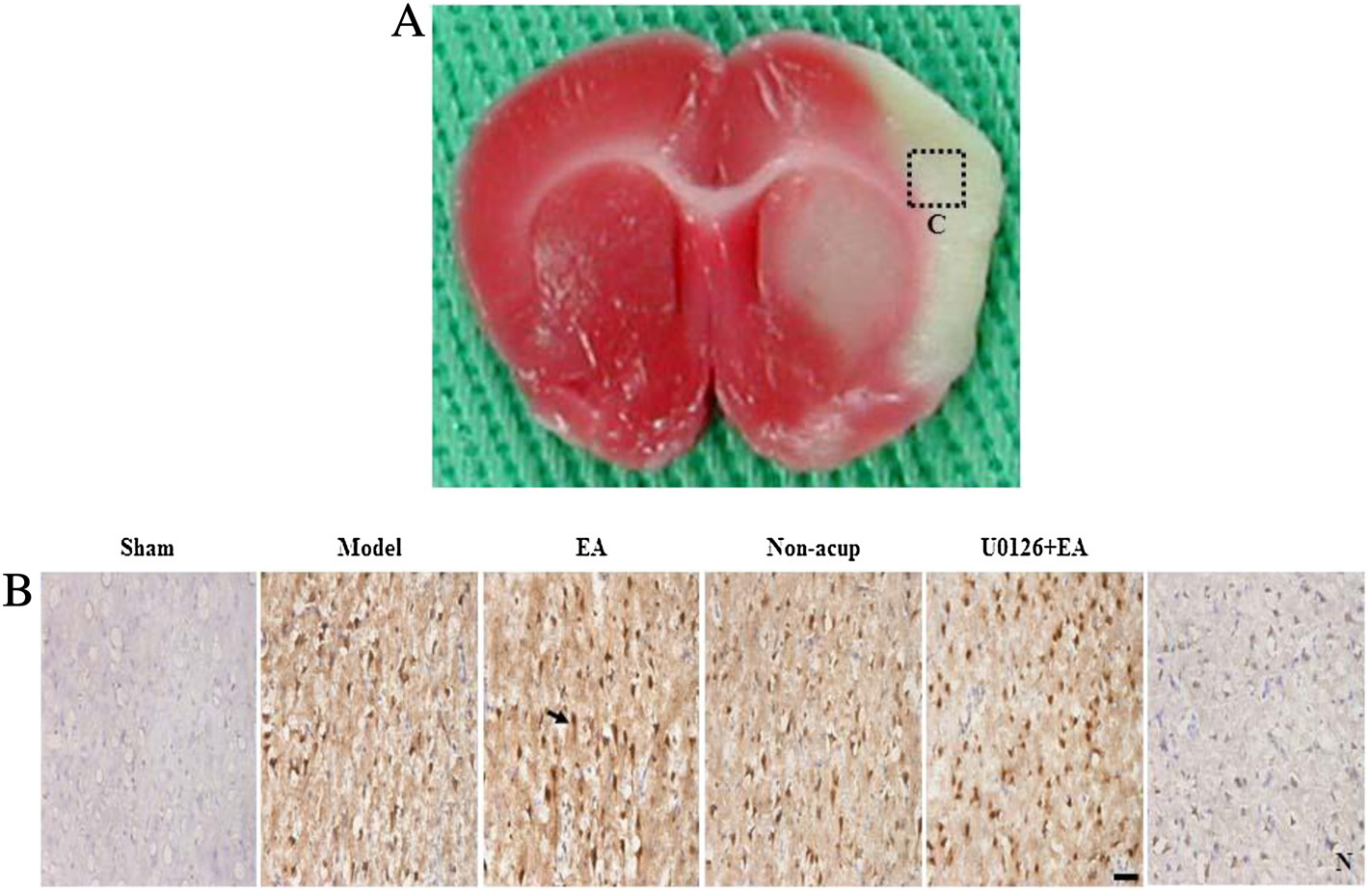
**Effect of EA at acupoints on BDNF expression in the ischemic cortex. (A)** Representative photograph showed a brain coronal section (TTC stain) from posterior bregma 0.92 mm. The dotted line square indicates the area of evaluation of immunopositive cells. C, the ischemic area of the cortex. Dotted line square = 1 mm^2^. **(B)** Representative photographs showed BDNF expression in the ischemic cortex of the sham, model, EA, non-acup, and U0126 + EA groups after 3 d of reperfusion. N, negative control stain. Arrow indicates a BDNF-positive cell. Scale bar = 50 μm.

**Figure 4:**
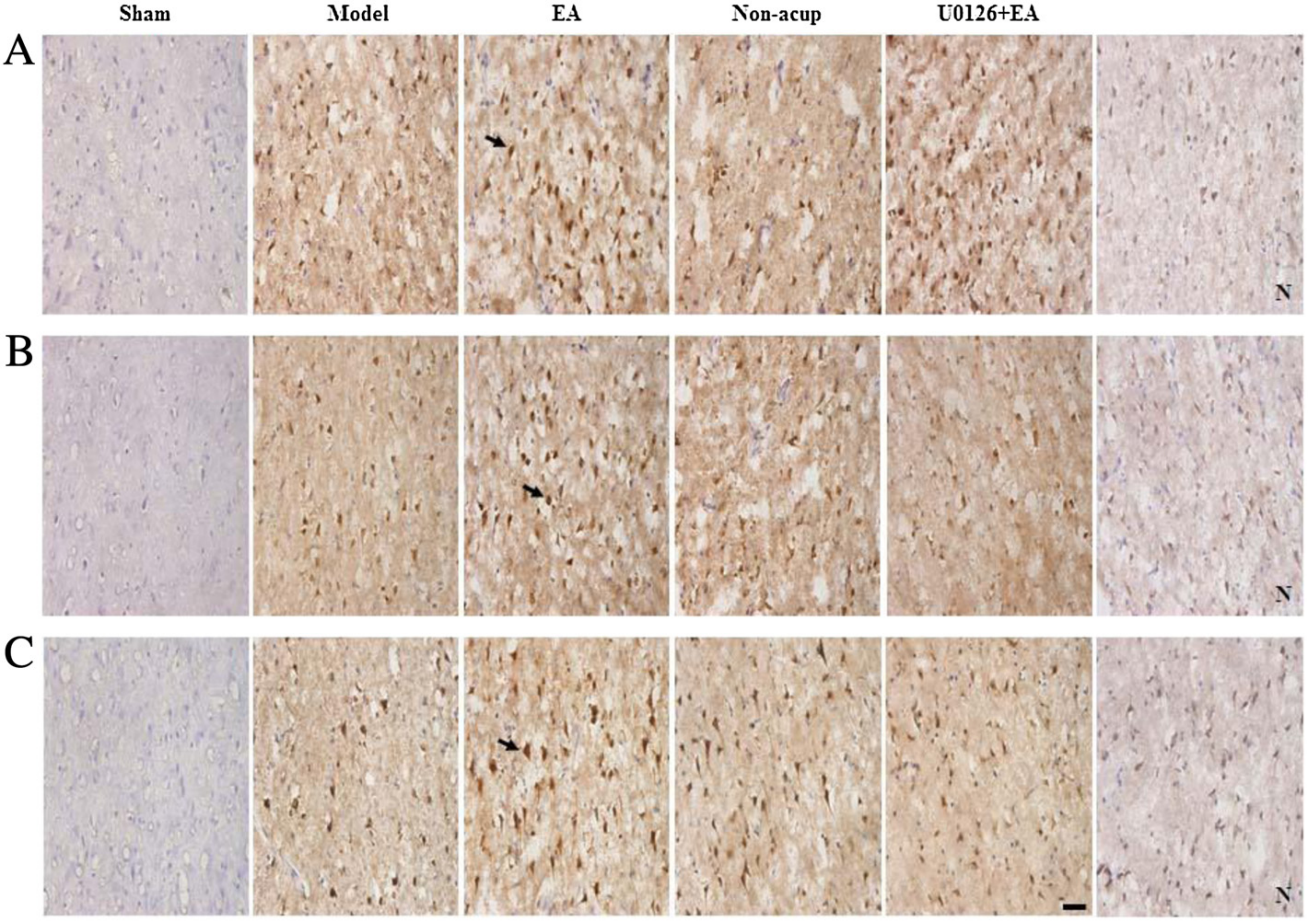
**Effects of EA at acupoints on pRaf-1, pMEK1/2, and pERK1/2 expression. (A)** Representative photographs showed pRaf-1, **(B)** pMEK1/2, and **(C)** pERK1/2 expression in the ischemic cortex of the sham, model, EA, non-acup, and U0126 + EA groups after 3 d of reperfusion. N, negative control stain. Arrows indicate immunopositive cells. Scale bar = 50 μm.

**Figure 5:**
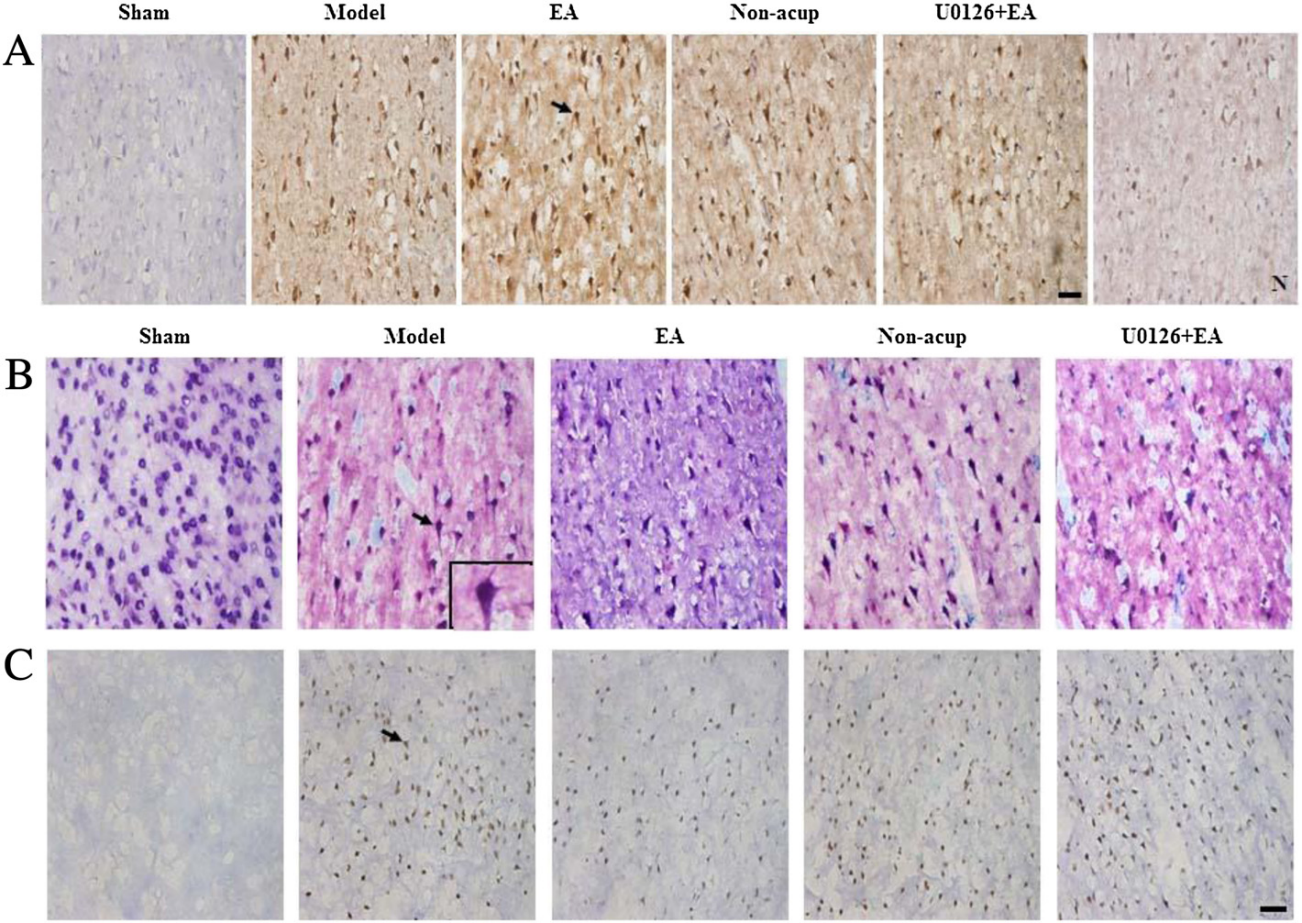
**Effects of EA at acupoints on pp90RSK, active caspase-3-NeuN, and TUNEL expression. (A)** Representative photographs showed pp90RSK expression, **(B)** active caspase-3 (red) colocalizing with NeuN (blue), and **(C)** TUNEL-positive cells in the ischemic cortex of the sham, model, EA, non-acup, and U0126 + EA groups after 3 d of reperfusion. N, negative control stain. Arrows in **(A)** and **(C)** indicate pp90RSK- and TUNEL-positive cells, respectively. Arrow in **(B)** indicates active caspase-3-NeuN double-labeled cells (purple), shown at higher magnification in the bottom right panel. Scale bar = 50 μm.

We evaluated BDNF-, pRaf-1-, pMEK1/2-, pERK1/2-, and pp90RSK-positive cells within the dotted line square of brain coronal sections (counts/1 mm^2^; Figure 3A). After 3 d of reperfusion, we observed a greater number of BDNF-, pRaf-1-, pMEK1/2-, pERK1/2-, and pp90RSK-positive cells in the ischemic cortex in the model, EA, non-acup, and U0126 + EA groups compared to the sham group (all *P* < 0.05; Figures 3B, 4A, B and C and 5A; Table 1). We also observed a significantly higher number of BDNF-, pRaf-1-, pMEK1/2-, pERK1/2-, and pp90RSK-positive cells in the ischemic cortex in the EA group compared to the model group (all *P* < 0.05; Figures 3B, 4A, B and C and 5A; Table 1). However, the levels of immunopositivity in the non-acup and model groups showed no significant differences (all *P* > 0.05; Figures 3B, 4A, B and C and 5A; Table 1). The numbers of BDNF- and pRaf-1-positive cells in the ischemic cortex were significantly higher in the U0126 + EA group than in the model group (both *P* < 0.05; Figures 3B and 4A; Table 1). However, the numbers of pMEK1/2-, pERK1/2-, and pp90RSK-positive cells in the U0126 + EA and model groups showed no significant differences (all *P* > 0.05; Figures 4B and C and 5A; Table 1). These results indicated that U0126 pretreatment did not influence BDNF or pRaf-1 positivity, but decreased pMEK1/2, pERK1/2, and pp90RSK positivity in the U0126 + EA group after 3 d of reperfusion.

**Table 1:** **Expression of BDNF-, pRaf-1-, pMEK1/2-, pERK1/2-, pp90RSK-, and TUNEL-positive cells (counts/1 mm**^**2**^**)**

	**Sham**	**Model**	**EA**	**Non-acup**	**U0126 + EA**
BDNF	0 ± 0	186 ± 46*	413 ± 60*#	178 ± 51*	393 ± 63*#
pRaf-1	0 ± 0	149 ± 39*	348 ± 55*#	187 ± 67*	338 ± 25*#
pMEK1/2	0 ± 0	165 ± 54*	274 ± 31*#	149 ± 52*	83 ± 54*
pERK1/2	0 ± 0	257 ± 52*	417 ± 36*#	219 ± 57*	198 ± 35*
pp90RSK	0 ± 0	261 ± 107*	497 ± 31*#	302 ± 98*	196 ± 50*
TUNEL	0 ± 0	365 ± 88*	209 ± 59*#	356 ± 80*	339 ± 79*

Mean ± SD (n = 5 or 6). Sham, sham group; Model, model group; EA, EA group; Non-acup, non-acup group; U0126 + EA, U0126 + EA group. *P < 0.05 vs. sham group; #*P* < 0.05 vs. model group.

### Effects of EA at acupoints on active caspase-3-NeuN costaining

Analysis of active caspase-3-NeuN costaining revealed numerous NeuN-positive cells (blue) in the sham group. Active caspase-3-positive cells (red) were predominant in the ischemic cortex in the model, non-acup, and U0126 + EA groups, whereas NeuN-positive cells were highly expressed in the EA group (Figure 5B). Cells displaying NeuN and active caspase-3 costaining (purple) were scattered in the ischemic cortex in the model, non-acup, and U0126 + EA groups. Staining for NeuN and active caspase-3 generally showed opposite patterns in the experimental groups (Figure 5B).

### Effects of EA at acupoints on the expression of TUNEL-positive cells

We observed increased TUNEL positivity in the ischemic cortex in the model, EA, non-acup, and U0126 + EA group (*P* < 0.05 vs. sham group; Figure 5C; Table 1) after 3 d of reperfusion. In the EA group, however, TUNEL positivity was reduced significantly compared with the model group (*P* < 0.05; Figure 5C; Table 1). The number of TUNEL-positive cells in the model, non-acup, and U0126 + EA groups showed no significant difference (*P* > 0.05; Figure 5C; Table 1).

### Effects of EA at acupoints on cytosolic expression of pMEK1/2, pERK1/2, pp90RSK, and pBad

**Figure 6:**
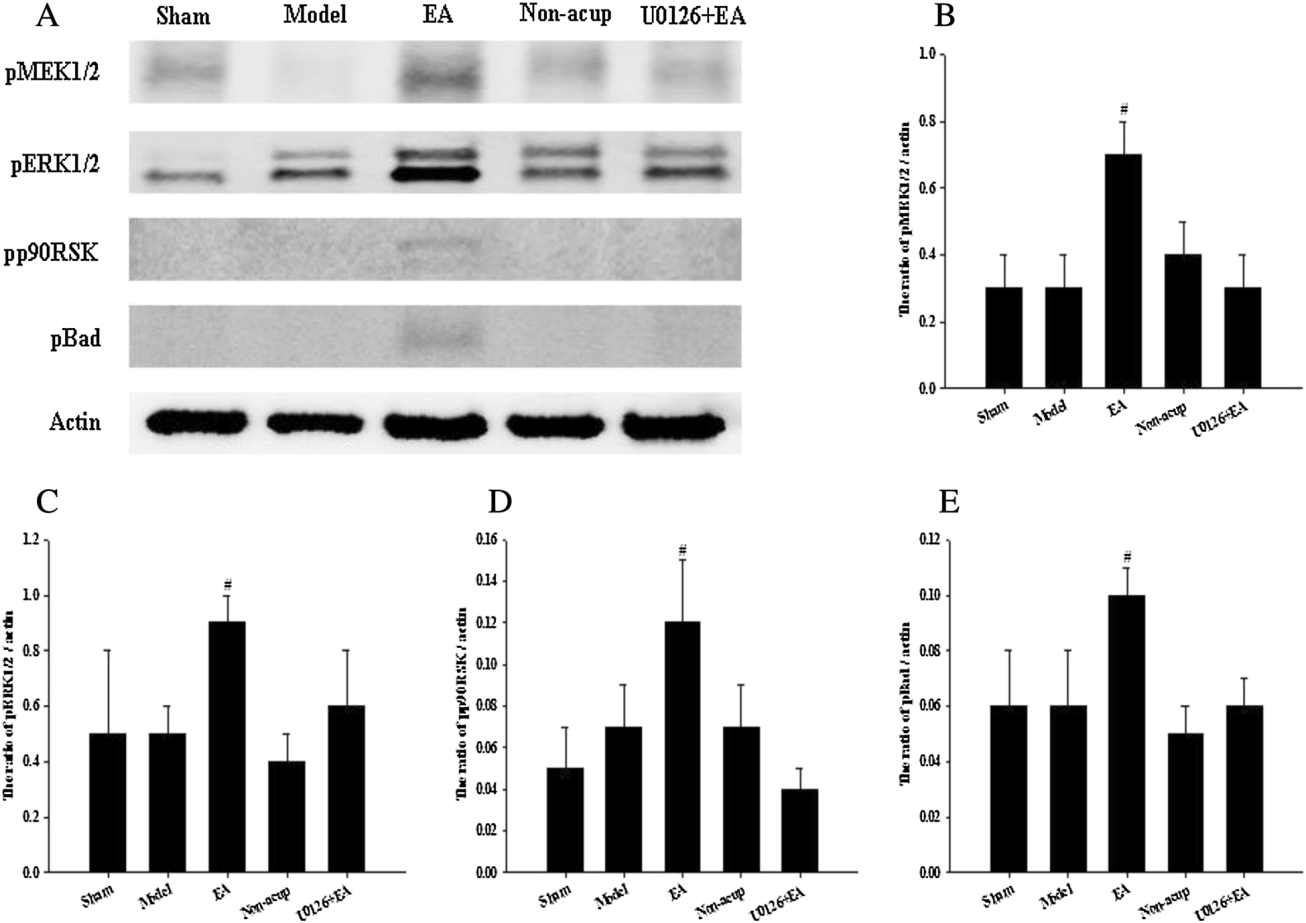
**Effects of EA at acupoints on cytosolic expression of pMEK1/2, pERK1/2, pp90RSK, and pBad. (A)** Representative western blot images showed the cytosolic expression of pMEK1/2, pERK1/2, pp90RSK, and pBad in the ischemic cortex in the sham, model, EA, non-acup, and U0126 + EA groups after 3 d of reperfusion. Actin was used as an internal control. The relative cytosolic expression of **(B)** pMEK1/2, **(C)** pERK1/2, **(D)** pp90RSK, and **(E)** pBad (n = 4) was assessed in the ischemic cortex in the sham, model, EA, non-acup, and U0126 + EA groups. Data are presented as mean ± SD. #*P* < 0.05 compared with the model group.

In western blot analysis, we observed that the cytosolic expression of pMEK1/2 in the ischemic cortex after 3 d of reperfusion showed no significant difference among the model, non-acup, and U0126 + EA groups (*P* > 0.05). However, cytosolic pMEK1/2 expression was significantly higher in the EA group than that in the model group (2.7-fold, *P* < 0.05; Figure 6A and B). We also evaluated the expression of pERK1/2, the downstream target of pMEK1/2, observing significantly higher cytosolic pERK1/2 expression in the EA group compared with the model group (1.8-fold, *P* < 0.05; Figure 6A and C). Cytosolic pERK1/2 expression among the model, non-acup, and U0126 + EA groups showed no significant difference (*P* > 0.05). Cytosolic pp90RSK expression was significantly higher in the EA group than in the model group (1.7-fold, *P* < 0.05; Figure 6A and D). However, cytosolic pp90RSK expression showed no significant difference among the model, non-acup, and U0126 + EA groups (*P* > 0.05). Cytosolic pBad expression was significantly higher in the EA group than in the model group (1.7-fold, *P* < 0.05; Figure 6A and E). However, cytosolic pBad expression showed no significant difference among the model, non-acup, and U0126 + EA groups (*P* > 0.05).

## Discussion

In this study, a 15-min period of MCAo consistently caused gross infarction after 3 d of reperfusion. This result was in accordance with those of previous studies, in which mild focal cerebral ischemia models showed markedly delayed infarct development after 72 h of reperfusion [2-4]. Our data also indicated that EA at acupoints, applied at 1 d after cerebral I/R injury and once daily for 2 consecutive days, effectively reduced cerebral infarct areas and neurological deficits, whereas EA at nonacupoints did not attenuate cerebral ischemic injury and behavioral deficits after 3 d of reperfusion. Previous studies have reported that preconditioning with repeated EA at the Baihui acupoint provided neuroprotective effects against focal cerebral ischemia in rats [20,28]. Our study findings further indicated that 2 repeated EA-like stimulations at Baihui and Dazhui acupoints, but not at nonacupoints, provided significant neuroprotection against cerebral I/R injury in a mild focal cerebral ischemia model.

Accumulating evidence has shown that BDNF plays an important role in brain development and plasticity, and that exogenous and endogenous BDNF promote synaptic plasticity and axon growth, which correlate positively with behavioral change and neurological recovery in transient cerebral I/R injury [29-31]. Studies have also shown that BDNF exerts neuroprotective effects against cerebral infarction by activating intercellular survival signaling pathways in transient MCAo in rats [10,16,32]. In our evaluations, we observed that EA at acupoints increased the expression of BDNF in the ischemic cortex significantly after 3 d of reperfusion. On the basis of these findings, we suggest that EA at acupoints exerted its neuroprotective effects against cerebral infarction and behavioral deficits in our mild MCAo model, at least partly, through the upregulation of BDNF expression.

Apoptosis is a prominent feature in mild focal cerebral ischemia and plays a crucial pathological role in the development of delayed infarction. Active caspase-3, which is a pivotal apoptotic executioner and causes cells to undergo nuclear condensation and DNA fragmentation, was increased significantly 24 h to 72 h postreperfusion [2]. Other studies have reported that the administration of apoptosis inhibitors 6 h postreperfusion exerted beneficial effects on cerebral I/R insults after 3 d or 14 d of reperfusion in mild MCAo [4,33]. In our TUNEL assays, the number of apoptotic cells showed marked increases in the ischemic cortex after 3 d of reperfusion, and EA at acupoints (initiated after 1 d of reperfusion) markedly reduced apoptotic activity in the ischemic cortex. A previous study has reported that NeuN, a marker of mature neurons, colocalized with apoptotic cells in the ischemic area 3 d after mild focal cerebral ischemia [34]. In our study, double staining for active caspase-3 and NeuN revealed that active caspase-3-labeling colocalized with relatively weak NeuN labeling, and markedly increased in the ischemic cortex after 3 d of reperfusion, consistent with changes in apoptosis. However, EA at acupoints markedly suppressed any increases in active caspase-3-labeling. In contrast, EA at acupoints effectively restored NeuN labeling through antigen retrieval. These results are consistent with those of the study by Cheng et al., which identified a negative feedback loop between caspase-3-dependent apoptosis and NeuN immunoreactivity in the model and caspase inhibitor-treated groups following cerebral I/R injury [35]. Our findings further indicated that EA at acupoints provides BDNF-mediated neuroprotection against cerebral I/R injury through inhibition of caspase-3-dependent neuronal apoptosis in the ischemic cortex after 3 d of reperfusion, and that posttreatment of EA at acupoints extends the effective time window for up to 24 h postreperfusion following mild MCAo.

Previous studies have well-described that BDNF promotes cortical neuron survival in response to ischemic insult through activation of the ERK1/2 signaling pathway, which includes Raf-1, MEK1/2, and ERK1/2 phosphorylation [10,36,37]. They have also shown that activation of the Raf-1/MEK1/2/ERK1/2 signaling pathway provides neuroprotective effects through the inhibition of neuronal apoptosis during focal cerebral ischemia [11,14,15]. The downstream target of the Raf-1/MEK1/2/ERK1/2 signaling pathway is p90RSK and a number of studies have proposed that pharmacologically selective activation of the Raf-1/MEK1/2/ERK1/2 signaling pathway elicits neuroprotective effects through the phosphorylation of p90RSK and Bad. Phosphorylated Bad binds to 14-3-3 to prevent the interaction between Bad and antiapoptotic proteins (Bcl-2 and Bcl-xL), which inhibits mitochondrial permeability transition pore formation and suppresses caspase-3-dependent apoptosis in permanent [11,38] and mild transient [39] MCAo models. However, it remains obscure whether BDNF-mediated neuroprotection resulting from EA stimulation involves phosphorylation of p90RSK and Bad following cerebral I/R injury. When evaluating the expression of molecules related to the ERK1/2 signaling pathway, we observed sparse pRaf-1, pMEK1/2, pERK1/2, and pp90RSK expression in the ischemic cortex after 3 d of reperfusion. However, EA at acupoints effectively increased the expression of these protein kinases in our mild transient MCAo model. Western blot analysis further showed that EA at acupoints effectively increased the cytosolic expression of pMEK1/2, pERK1/2, pp90RSK, and pBad in the ischemic cortex after 3 d of reperfusion. Our results suggested that EA at acupoints elicits BDNF-mediated neuroprotective action against caspase-3-dependent neuronal apoptosis through activation of the Raf-1/MEK1/2/ERK1/2 signaling pathway, and that the ERK1/2 signaling pathway-mediated neuroprotective effects of EA at acupoints can be further attributed to the phosphorylation of p90RSK and Bad in the ischemic cortex after 3 d of reperfusion following mild MCAo.

During cerebral ischemia progression, the survival signaling cascades activated by neuroprotective agents include the PI3K and MAPK/ERK1/2 signaling pathways, which can cause cross-reactions and prevent apoptosis [15,37]. Several reports have described that EA posttreatment elicits neuroprotective action against ischemic insults through the activation of the PI3k signaling pathway after 1 d of reperfusion in mild [24,25] and moderate [23] focal cerebral ischemia models. One study done by Du et al., has shown that EA pretreatment elicited neuroprotective effects through activation of the ERK1/2 signaling pathway after 1 d of reperfusion in a severe MCAo model [20]. These results indicated that EA treatment can potentially provide neuroprotection against cerebral I/R injury by activating PI3K and ERK1/2 signaling pathways in MCAo models. Previous studies have also reported that pharmacological activators of the ERK1/2 signaling pathway elicit neuroprotection through the upregulation of BDNF expression in cerebral ischemia models [9,40]. Therefore, to gain further insight into the possible role of the ERK1/2 signaling pathway in BDNF-mediated neuroprotection induced by EA at acupoints, we examined the effects of the MEK1/2 inhibitor U0126, which can inhibit activation of ERK1/2 by inhibiting MEK1/2 and eradicate ERK1/2 signaling pathway-mediated neuroprotective effects in transient MCAo [20,39]. In our evaluations, we observed that in the U0126 + EA group, administration of U0126 30 min prior to the onset of EA at acupoints fully eradicated the neuroprotective effects of EA at acupoints against cerebral infarction, neurological deficits, and caspase-3-dependent neuronal apoptosis after 3 d of reperfusion. During further analysis of the expression of ERK1/2 signaling-related protein kinases and BDNF, we observed that pretreatment with U0126 abrogated the upregulating effects of EA at acupoints on cytoplasmic pMEK1/2, pERK1/2, pp90RSK and pBad expression. However, U0126 pretreatment did not affect the upregulating effects of EA at acupoints on upstream kinase pRaf-1 or BDNF expression. Based on these findings, we propose that EA at acupoints (initiated 1 d postreperfusion) upregulated BDNF expression, which subsequently upregulated the expression of Raf-1. In addition, U0126 pretreatment eradicated the ERK1/2 signaling pathway-mediated neuroprotection induced by EA at acupoints, confirming that in our mild MCAo model, activation of the ERK1/2 signaling pathway, and subsequent phosphorylation of p90RSK and Bad, induced BDNF-mediated neuroprotection against caspase-3-dependent neuronal apoptosis after 3 d of reperfusion. To our knowledge, this is the first study to show that EA at acupoints induces BDNF-mediated neuroprotection against apoptosis through phosphorylation of ERK1/2/p90RSk/Bad pathway in the model of mild transient focal cerebral ischemia.

## Conclusion

In this study, EA at acupoints, initiated 1 d postreperfusion, effectively upregulated BDNF expression to provide BDNF-mediated neuroprotection against neuronal apoptosis through phosphorylation of the Raf-1/MEK1/2/ERK1/2/p90RSK/Bad signaling cascade after 3 d of reperfusion. Our data suggest that EA at acupoints could potentially provide a therapeutic strategy to extend the time window in mild cerebral I/R injury, and warrants further investigation for future clinic application.

## Abbreviations

EA: Electroacupuncture; I/R: Ischemia-reperfusion; BDNF: Brain-derived neurotrophic factor; ERK: Extracellular signal-regulated kinase; MCAo: Middle cerebral artery occlusion; pRaf-1: Phospho-Raf-1; MAPK: Mitogen-activated protein kinase; MEK1/2: MAPK/ERK kinase1/2; pMEK1/2: Phospho-MEK1/2; pERK1/2: Phospho-ERK1/2; p90RSK: 90 kDa ribosomal S6 kinase; pp90RSK: Phospho- p90RSK; NeuN: Neuronal nuclei; TrkB: Tyrosine kinase B; PI3K: Phosphatidylinositol 3-kinase.

## Competing interests

The authors declare that they have no competing interests.

## Authors’ contributions

CHL participated in the design of the study. CYC and SYS performed research, analyzed data and wrote the manuscript. JGL, NYT and STK helped to draft the manuscript. All authors read and approved the final manuscript.

## Pre-publication history

The pre-publication history for this paper can be accessed here:

http://www.biomedcentral.com/1472-6882/14/92/prepub
